# Quality of Life in University Students with Diabetes Distress: Type 1 and Type 2 of Diabetes Differences

**DOI:** 10.1155/2022/1633448

**Published:** 2022-06-24

**Authors:** Samah A. Moawd

**Affiliations:** ^1^Department of Health and Rehabilitation Sciences, College of Applied Medical Sciences, Prince Sattam bin Abdulaziz University, Al-Kharj 11942, Saudi Arabia; ^2^Department of Physical Therapy for Cardiovascular/Respiratory Disorders and Geriatrics, Faculty of Physical Therapy, Cairo University, Giza 12613, Egypt

## Abstract

**Background:**

This study constitutes a preliminary trial to clarify the relationship between quality of life (QoL) and diabetes distress (DD in patients with diabetes mellitus (DM) by comparing patients with type 1 and type 2 diabetes.

**Methods:**

A cross-sectional study of university students with diabetes (type 1 and type 2) diabetes. One hundred sixty-six students were assigned to participate in this study. A self-report questionnaire on demographic and clinical parameters was taken. Rating worries and anxieties related to diabetes were evaluated using the diabetes distress scale-17, and quality of life was tested using SF-36 v2.

**Results:**

No significant differences were observed in the level of DD according to sociodemographics in type 1 DM (T1DM) and type 2 DM (T2DM) (*p* > 0.05). The mean scores for Physical Component Summary (PCS) and Mental Component Summary (MCS) and six subscales of the SF-36 v2 demonstrated no significant differences between T1DM and T2DM (*p* > 0.05). High scores of diabetes distress were independently associated with lower glycemic control for students with both types of diabetes. Likewise, high scores of distress were associated with lower PCS (*p* < 0.05). Additionally, the results showed that high scores of diabetes distress were associated with lower MCS (*p* < 0.05).

**Conclusions:**

University students with diabetes showed a high level of DD with no significant differences between both types of diabetes; this consequently affects all components of QoL. Psychological support is the better choice for those students for better health and future career.

## 1. Introduction

Diabetes mellitus is definitely one of the greatest medical challenges in this century. It marks T2DM or type 1 T1DM whether they inject insulin or not [1]. Diabetics are at high risk of developing psychological problems such as sadness, nervousness, eating disorders, dementia, diabetes distress (DD), psychological insulin resistance, and persistent fear of hypoglycemia [2]. All these complications are associated with a decrease in diabetic patients' activities to maintain or improve their own health and the outcomes associated with their disease [3]. Moreover, these factors have a negative impact on quality of life as the physical and emotional complications of diabetes are interrelated [4].

DD is a concern related to diabetes treatments, social support and accessibility to care, and emotional distress and anxiety. This often includes worries about diagnosis, complications, and management requirements (blood glucose testing, diet, and exercise) or treatment, leading to poor diabetes adjustment and problems with self-care; it is a nonpsychiatric condition that requires special attention for better mental, emotional, and physical health [5]. Its high prevalence among adults is associated with poor diabetes control and management, and it is also strongly associated with diabetes self-efficacy and quality of life [6].

There was a negative correlation between DD and health-related quality of life (HRQoL) [7]; comparable results were documented by Carper et al. who observed the association between diabetes burden and quality of life and suggested that poorer quality of life was associated with poor glycemic control and high DD; therefore, earlier detection and treatment of DD was recommended [8].

University places the individual in a new environment characterized by easy detachment from family, new social relationships, and increased independence and responsibility. For diabetics, university is considered a high-risk environment. In many of the existing accounts of students with diabetes, university is seen as an environment where students can become “out of control.” Diabetic students do not know how to deal with the challenges of university life [9]. However, there is a dearth of studies on the relationship between DD and health-related quality of life (HRQoL), self-efficacy, and disease control in university students with diabetes [10]. The current study was conducted to assess the extent of diabetes burden in university students with diabetes and its associated factors such as glycemic control and quality of life, hypothesizing that DD may have negative effects on glycemic control and quality of life in these students.

## 2. Methodology

A cross-sectional study of university students with diabetes was conducted for the duration of the study from January 2020 to October 2020. The ethical clearance was accomplished by the local institutional review board of the physiotherapy department (No.: RHPT/020/053). All procedures were achieved in compliance with the ethical standards of the 1964 Declaration of Helsinki and its updates. Data were collected through interviews from patient records in Riyadh Hospitals, Saudi Arabia. The selected patients were from different places, health centers, diabetic centers, and different places in the middle province to be treated appropriately.

One hundred eighty- seven students were screened for the study, 21 (10 type 1 and 11 type 2) did not complete the questionnaire. One hundred sixty-six (80 type 1 and 86 type 2 diabetes) were included.

The cases were students with type 1 or type 2 diabetes who agreed to participate in the study and recorded HbA1c results for the last 3 months of the session in their files along with height and weight. Confused, frail, or disabled students were excluded from the study.

### 2.1. Demographic and Clinical Variables

Students were given a self-report questionnaire on age, sex, duration of diabetes, and exercise. Clinical data include hypertension (SBP ≥ 130 mmHg or DBP ≥ 80 mmHg) and dyslipidemia (LDL cholesterol 2.6 mmol/L or triglycerides (TG) 1.7 mmol/L or HDL cholesterol 1.1 mmol/L) and diabetic complications such as retinopathy, nephropathy, diabetic foot, ischemic heart disease, and cerebrovascular disease. A data set from the patient's record that includes HbA1-cl level, blood pressure, lipids, and medications was used. In addition, a sleep time calculation consisted of night sleep time and nap time (30-120 min) [11].

### 2.2. Diabetes Distress Scale-17

The DDS-17 captures diabetes-related distress by rating worries and anxieties related to diabetes during the past month on a Likert scale from 1 (not a problem) to 6 (a very serious problem). There are four subscales of the DDS; emotional distress (EB), physician stress (PD), regimen stress (RD), and interpersonal diabetes (ID) stress were reported in addition to the total score. A total score of less than 2 indicates low to no distress, 2 to 2.9 indicates moderate distress, and 3 or higher indicates high distress [12]. An Arabic version of the DDS-17 was created by a bilingual expert and validated by two other bilingual experts with internal consistency for the entire 17 items (Cronbach′s alpha = 0.877) [13].

### 2.3. SF-36 v2

This abbreviated version of the SF-36 Health Questionnaire quality of life assessment consists of 36 questions covering eight conceptual domains: physical functioning, role limitations due to physical health, physical pain, general health perception, vitality, social functioning, role limitations due to emotional problems, and mental health. For each dimension, the scores are summed, with 0 (worst health) to 100 (best health). Two summary measures were combined from SF-36 v2: the Physical Component Summary (PCS) and the Mental Component Summary (MCS), which are calculated using a standard methodology. There is strong evidence for the reliability and validity of the instrument, and it is the largest thoroughly tested and widely accepted measure in many countries [14].

### 2.4. Sample Size Estimation

Using the G-Power software, V3.0.10 (Neu-Isenburg, Germany) was used to find the proper sample size and to reverse the effect of type II error, mean difference, and standard deviation of total distress score (MD = 2.17 and SD = 0.65) that were accomplished from a pilot study consisting of20 patients. Pretend an alpha error of 5% and the anticipated power of 90%, and an overall sample of 134 participants (67 subjects for each group) were required, and the estimated sample size was 166, accounting for an approximate dropout rate of 20%.

### 2.5. Statistical Analysis

Calculation of mean ± standard deviation (SD) was done using Student's *t*-test, one-way analysis of variance (ANOVA) was used to test for changes in means between groups, while the Mann-Whitney *U* test was used to compute quality of life according to treatment type. Linear regression analysis was performed between Qol scores and DDS-17. All analyses were performed using SPSS version 16.0 (SPSS Inc, Chicago, IL). A *p* value of less than 0.05 was considered significant.

## 3. Results

**Figure 1:**
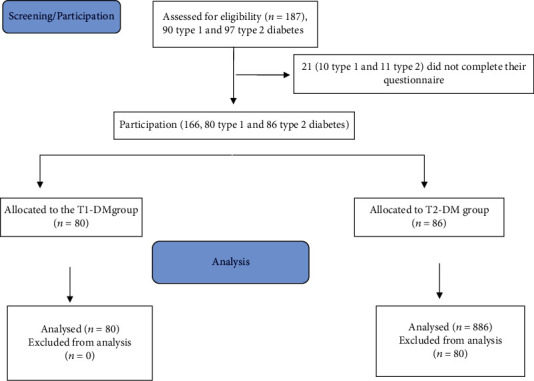
The CONSORT flow diagram of the study.

Of 187 (90 type 1 and 97 type 2) eligible diabetic students, 21 (10 type 1 and 11 type 2) did not complete their questionnaire and were therefore dropped from the analysis, and 166 (80 type 1 and 86 type 2) completed the study (Figure 1). Their sociodemographic and clinical data are shown in (Table 1); there were nonsignificant differences in all sociodemographic and clinical data except diabetes duration between the two types of diabetes except for diabetic duration, BMI, and medication; there were significant differences between both groups (*p* = 0.001). The mean age of patients was 20.73 yrs; BMI was 24.31 for T1DM and 29.31 for T2DM, and diabetes duration was 14.16 for T1DM and 10.17 for T2DM. 87.5% of T1DM students were based on insulin in their medication, while 73.3% of T2DM students were based on oral hypoglycemic medication.

### 3.1. Demographic, Clinical Parameters, and Diabetes Distress Level

The level of DD according to sociodemographics is shown in (Table 2); there was no significant difference between the different age groups and gender in both groups (*p* > 0.05), while there were highly significant differences in the level of diabetes distress (*p* = 0.001) in the two groups in terms of BMI (normal, overweight, and obese), exercise (active or inactive), HbA1c, % (or ≥7), diabetic complication (present or absent), and medication used (diet and exercise, OHA, insulin, and insulin and oral medication), with more stress in students who were obese (2.98 ± 0.51 and 3.1 ± 0.49), inactive (2.28 ± 0.69 and 2.61 ± 0.59), uncontrolled HbA1c levels (2.27 ± 0.73 and 2.47 ± 0.63), with diabetic complications (2.4 ± 0.9 and 2.86 ± 0.8), and dependent on insulin and oral medications (2.9 ± 0.6 and 2.97 ± 0.8) for T1DM and T2DM groups, respectively.

### 3.2. Quality of Life Is Related with Diabetes Distress

Table 3 shows an example of SF-36 v2 outcome analysis comparing the quality of life between two types of diabetes (T1DM and T2DM). There were no significant changes between the two groups in physical functioning (*p* = 0.17), role limitations due to physical health (*p* = 0.22), physical pain (*p* = 0.13), role limitations due to emotional problems (*p* = 0.31), general health perception (*p* = 0.47), vitality (*p* = 0.54), social functioning (*p* = 0.63), and mental health (*p* = 0.29). Students with T2DM had lower PCS and MCS scores (46.46 and 45.3) compared to T1DM students, but no significant difference was found when comparing PCS and MCS (*p* = 0.44, 0.15) in both groups.

The linear regression models presented in Table 4 showed the associations between diabetes distress and age, BMI, exercise, and glycemic control as measured by HbA1C levels and quality of life (PCS and MCS) in students with T1DM and T2DM.

Higher DD scores were significantly associated with high BMI (standardized *β* = .283, *p* = .04; standardized *β* = 0.337, *p* = 0.01) and also with less exercise (standardized *β* = −0.217, <.001; standardized *β* = 0.274, *p* = <.001), but not with age of the students (standardized *β* = .024, *p* = 0.25; standardized *β* = 0.032, *p* = 0.34) in T1DM and T2DM, respectively.

High scores of diabetes distress were independently associated with lower glycemic control for students with T1DM and T2DM, respectively (standardized *β* = .343, *p* = .027; standardized *β* = 0.258, *p* = 0.044). Similarly, high scores of distress were associated with lower PCS (standardized *β* = 0.641, *p* = .001; standardized *β* = 0.472, *p* = .001). In addition, the results showed that high scores of diabetes distress were associated with lower MCS (standardized *β* = .0.442, *p* = 0.001; standardized *β* = 0.417, *p* = .001).

## 4. Discussion

This cross-sectional study is aimed at assessing the prevalence of diabetes distress in T1DM and T2DM university students and its impact on quality of life. We hypothesized that diabetes distress might have a worthless impact on the quality of life in university students with diabetes and this effect is the same in both T1DM and T2DM. The results of the study confirmed our hypothesis and showed that the university students with T1DM and T2DM had high diabetes distress, and it negatively affected the quality of life of these students.

In the current study, the prevalence of diabetes stress according to sociodemographic and clinical parameters among T1DM and T2DM students showed a nonsignificant difference in almost all parameters except BMI, diabetes duration, and medication. Also, no age or sex differences were observed in this study with respect to diabetes stress in the two study groups. Beverly et al. [15] agreed with the current study, and they noted that no sex differences were observed in this study, although diabetes stress was associated with positive screening for major depression.

There was a significant change regarding the influence of exercise on DD; these results were similar to those shown by Perrin et al. [16]. They assessed the level of psychological distress and observed the related factors through a cross-sectional study of type 2 diabetic patients and found that inactive individuals developed a high diabetes distress score. Moreover, in our study, there was a significant difference in that diabetes-related distress was related to HbA1c. In line with the current study, a study on Japanese investigated the association between Problem Areas in Diabetes (PAID) Scale scores and glycemic control with diabetes therapy and concluded that DRD was associated with poor glycemic control [17]. Another study on Korean Americans with type 2 diabetes mellitus examined the relationships of DRD and depressive symptoms with glycemic control and suggested that DRD was positively correlated with HbA1c levels, whereas depressive symptoms were not correlated with glycemic control [18]. In contrast, a study of patients with type 2 diabetes mellitus in China [16] did not find that diabetes-related stress was related to HbA1c levels as they assessed the level of psychological stress and associated factors in T2DM.

Zhou et al. [12] found that the effect of BMI on diabetes stress levels was statistically significant. Also, another study found a significant difference in the effect of BMI on DD in adults with type 2 diabetes [19]. These results were consistent with our findings. According to Perrin et al. [16], DDS and EB were associated with poorer sleep time and lower self-efficacy and recommended interventions to improve sleep are needed, after evaluating the level of psychological distress and examining the associated factors. The reason for the association between diabetes stress and sleep is that poor sleep could lead to insulin resistance and be the cause of poor glycemic control [20], thus diabetes stress. The results of this study are in agreement with our study. Moreover, poorer sleep and associated psychological distress negatively affect the quality of life of patients with type 2 diabetes mellitus [21].

Moreover, the results of the present study revealed that there was a significant difference between different medications and diabetes stress, which also significantly affected diabetic complications in T1DM and T2DM groups. A high percentage (78.75%) of T1DM students relied on insulin, and among T2DM students, 40.7% relied on oral medications and 34.9% relied on insulin and oral medications. The findings of the current study are supported by the result of another study that found that there was a high incidence of diabetes distress among adults with T2DM and that diabetics who relied on insulin therapy were associated with overall diabetes distress, but in contrast, this study found that there was no direct association with diabetic complications [22].

As an aside, a study illustrating the factors influencing diabetes-related emotional distress with various medications found that patients who were dependent on insulin treatment were exposed to higher levels of DRD than those treated with oral hypoglycemic agents alone or with lifestyle modification alone [23]; all of these findings come in agreement with the current study. This may be related to the fact that insulin treatment by injection [24] is associated with poor glycemic control [25] and more severe complications; in addition, insulin therapy is more expensive than other types of medication [23].

The current study found that students are dependent on two types of medications; as insulin and oral hypoglycemic medications are higher diabetes burdens than other types of medications, this comes in agreement with Perrin et al. [16] who found that insulin and more oral hypoglycemic medications are exposed to higher DDS. This is due to the more complicated medication regimens, higher energy expenditure, and higher costs. In addition, the results of the current study are consistent with those of Fisher et al. [18] who found an association between diabetes burden and distress, with insulin being significantly associated with higher distress.

First, stress may be directly related to glycemic control through its effect on the neuroendocrine system and sympathetic nervous system, which lead to the release of stress hormones. Stress hormones increase glucose production in the liver, inhibit insulin secretion in the pancreas, and/or decrease the insulin response to glucose. In other words, it can directly alter blood glucose levels. A second explanation for why stress negatively affects glycemic control is that stress can indirectly affect glycemic control by distracting from self-care behaviors. It is also possible that the 2 pathways are linked [23, 24].

In the current study, there were no significant changes between the two groups in all domains of the quality of life questionnaire (SF-36 v2). In a study by Helgeson et al. [25], it was found that almost all adults with T2D mediated good overall HRQoL. However, DD showed poor effects on a variety of HRQoL domains. To improve HRQoL using an appropriate and simple psychological intervention, they examined the determinants of HRQoL, specifically the association between DD and HRQoL, taking into account sociodemographic-clinical variables, including depressive symptoms (DS), and showed that DS rather than DD had a more consistent, negative, and independent effect across all domains of HRQoL. Another study, consistent with the current study, examined the effect of diabetes burden on QoL and related clinical characteristics in Mexican T2DM patients and showed that increased diabetes burden was associated with decreased QoL [26]. Therefore, it is important to reduce factors associated with more emotional distress that affect patients with diabetes.

A systematic review examined the prevalence of DD; demographic, clinical, behavioral, and psychosocial associations of DD, and mediations reducing DD in adolescents with type 1 diabetes and found that DD was highly associated with depressive symptoms and negatively associated with diabetes-specific quality of life (QoL) [27]. There were prevalent and close associations between the domains of HRQoL and emotions (DRD and DS) in T2D adults [28]. Previous studies reported that diabetes symptoms were more likely to affect QoL than DRD in T2D adults and older people with diabetes [6, 11].

This study includes a relatively large sample with a high response rate and also represents the study population in terms of sociodemographic characteristics for the study area. Another important strength of the present study is the use of a validated and specific measure of DRD. The limitation of the study is that it is important to distinguish between general QoL and disease-specific HRQoL, as patients' needs might go unnoticed if general well-being or QoL is the only outcome measured and if it seems to be good.

## 5. Conclusion

University students with diabetes showed DD with no significant differences between T1DM and T2DM; this diabetes-related burden affects the quality of life; this requires additional studies to assess and prevent the long-term negative effects of DD on QoL associated with the progression of this disease. Psychological intervention could be a priority for these students to reduce the impact of diabetes stress and its associated consequences to improve their self-efficacy and quality of life.

## Figures and Tables

**Table 1:** The difference in demographic and clinical parameters of both groups.

Parameter	T1DM (*n* = 80)	T2DM (*n* = 86)	*p* ^∗^
	Mean ± SD	Mean ± SD	
Age, yr	20.03 ± 5.04	21.15 ± 3.95	0.137
Gender (M/F)	36/42	38/48	0.15
BMI (kg/m^2^)	24.31 ± 6.12	29.31 ± 5.72	<0.001^∗^
Diabetes duration, yr	14.16 ± 3.9	10.17 ± 2.19	<0.001^∗^
Sleep time	9.92 ± 2.32	9.62 ± 3.07	NS
LDL-C, mmol/L	142.2 ± 47.2	137.4 ± 38.9	0.131
HDL-C, mmol/L	42.64 ± 5.6	45.1 ± 6.7	0.125
TG, mmol/L	141.71 ± 35.9	136.2 ± 35.1	0.41
SBP (mmHg)	120.2 ± 7.1	117.3 ± 7.4	0.322
DBP (mmHg)	74.2 ± 6.3	75.8 ± 4.3	0.187
HbA1c, %	7.7 ± 1.4	7.46 ± 1.1	0.091
Medication			
Diet & exercise	5 ± 6.3	11 ± 19.7	<0.001^∗^
OHA	2 ± 1.3	63 ± 53.7	
Insulin	70 ± 82.9	2 ± 2.3	
Insulin and oral medication	3 ± 2.7	10 ± 7.9	

BMI: body mass index; LDL-C: low-density lipoprotein cholesterol; HDL-C: high-density lipoprotein cholesterol; TG: triglyceride; SBP: systolic blood pressure; DBP: diastolic blood pressure; HbA1c: the hemoglobin A1c; OHA: oral hypoglycemic agent. ^∗^Significant at 0.05 level.

**Table 2:** Sociodemographic and clinical parameters and level of diabetes distress.

Parameter		T1DM			T2DM		
		Number (%)	Total distress score Mean ± SD	*p* ^∗^	Number (%)	Total distress score Mean ± SD	*p* ^∗^
Age, yr	<20	44 (55)	2.19 ± 0.3	NS	48 **(**55.8)	2.22 ± 0.4	0.631
	≥20	36 (45)	2.81 ± 0.7		38 (44.2)	2.93 ± 08	
Gender	Male	39 (48.8)	2.13 ± 0.31	NS	40 **(**46.5)	2.27 ± 0.43	0.339
	Female	41 (51.2)	2.26 ± 0.45		46 **(**53.5)	2.3 ± 0.47	
BMI (kg/m^2^)	Normal	57 (62.3)	2.34 ± 0.7	<0.001^∗^	7 **(**8.2)	2.04 ± 0.7	<0.001^∗^
	Overweight	17 (21.3)	2.04 ± 0.2		49 **(**56.9)	2.44 ± 0.12	
	Obese	6 (7.4)	2.98 ± 0.51		30 **(**34.9)	3.1 ± 0.49	
Sleep time	<8 hr	33 (41.2)	2.67 ± 0.45	<0.001^∗^	37 **(**43.02)	2.91 ± 0.41	<0.001^∗^
	≥8 hr	47 (58.8)	1.97 ± 0.39		49 **(**56.98)	2.09 ± 0.42	
Exercise	Active	34 (42.5)	2.06 ± 0.71	<0.001^∗^	25 **(**29.06)	2.16 ± 0.53	<0.001^∗^
	Not active	46 (57.5)	2.28 ± 0.69		61 **(**70.93)	2.61 ± 0.59	
HbA1c, %	<7	30 (37.5)	1.97 ± 0.46	<0.001^∗^	27 **(**31.4)	2.09 ± 0.66	<0.001^∗^
	≥7	50 (62.5)	2.27 ± 0.73		59 **(**68.6)	2.47 ± 0.63	
Diabetic complication	No	42 (52.5)	2.08 ± 0.8	<0.001^∗^	45 **(**52.3)	1.68 ± 0.7	<0.001^∗^
	Yes	38 (47.5)	2.4 ± 0.9		41 **(**47.7)	2.86 ± 0.8	
Medication	Diet and exercise	10 (12.5)	1.78 ± 0.7	<.001^∗^	15 (17.5)	2.58 ± 0.4	<0.001^∗^
	OHA	1 (1.2)	2.01 ± 0.8		35 (40.7)	2.31 ± 0.8	
	Insulin	63 (78.8)	2.66 ± 0.5		6 **(**6.9)	2.9 ± 0.6	
	Insulin and oral medication	6 (7.5)	3.01.9 ± 0.6		30 **(**34.9)	3.03 ± 0.8	

BMI: body mass index; HbA1c: the hemoglobin A1c; OHA: oral hypoglycemic agent.

**Table 3:** The difference in quality of life between T1DM and T2DM students.

SF-36 v2 domains	T1DM (*M* ± SD)	T2DM (*M* ± SD)	*p* ^∗^
Physical functioning	73.18 ± 19.02	72.30 ± 20.13	0.17
Role physical	78.29 ± 24.62	68.22 ± 28.5	0.22
Bodily pain	70.27 ± 20.70	71.09 ± 21.8	0.13
PCS	49.81 ± 17.15	46.46 ± 18.01	0.44
Role emotional	69.05 ± 23.23	70.90 ± 20.09	0.31
General health perceptions	63.60 ± 20.61	61.03 ± 22.75	0.47
Vitality	70.30 ± 20.30	71.14 ± 28.04	0.54
Social functioning	70.34 ± 20.06	71.15 ± 20.04	0.63
MCS	47.80 ± 19.14	45.3 ± 20.11	0.15

PCS: Physical Component Summary; MCS: Mental Component Summary; ^∗^significant at 0.05 level.

**Table 4:** Linear regression between diabetes distress and age, BMI, exercise glycemic control, Physical Component Summary, and Mental Component Summary in T1DM and T2DM students.

Variable	DM	*R* ^2^	Standardized coefficient	*p* ^∗^
Age	T1DM	0.12	0.024	0.25
	T2DM	0.11	0.032	0.34
BMI	T1DM	0.14	0.283	0.04
	T2DM	0.17	0.337	0.01
Exercise	T1DM	0.21	-0.217	<.001
	T2DM	0.20	-0.274	<.001
GC	T1DM	0.18	0.343	0.027
	T2DM	0.13	0.258	0.074
PCS	T1DM	0.52	-0.641	<.001
	T2DM	0.64	-0.472	<.001
MCS	T1DM	0.46	-0.442	<.001
	T2DM	0.44	-0.317	<.001

BMI: body mass index; GC: glycemic control; PCS: Physical Component Summary; MCS: Mental Component Summary; ^∗^significant at 0.05 level.

## Data Availability

Regarding manuscript 1633448, the data involved is available from the corresponding author upon request and privacy-related parts of the patient will not be provided.
